# A comparative analysis of the Irish post-graduate geriatric medicine training scheme with the European post-graduate curriculum in geriatric medicine

**DOI:** 10.1007/s41999-023-00745-6

**Published:** 2023-01-13

**Authors:** Robert Murphy, Christine McCarthy, Catriona Reddin, Michelle Canavan, Clodagh O’Dwyer, Martin Mulroy, Martin O’Donnell

**Affiliations:** 1 Department of Geriatric & Stroke Medicine, Galway University Hospital, Galway, Ireland; 2 HRB Clinical Research Facility, University of Galway, Galway, Ireland; 3 Department of Geriatric Medicine, St. Vincent’s University Hospital, Dublin, Ireland; 4 Department of Geriatric Medicine, Our Lady of Lourdes Hospital, Drogheda, Louth, Ireland; 5 Department of Medicine, Royal College of Surgeons in Ireland, Dublin, Ireland

**Keywords:** Geriatric medicine training, Geriatric medicine curriculum, Post-graduate training, International geriatric medicine comparisons

## Abstract

**Purpose:**

Minimum training recommendations to become a specialist geriatrician in the EU have been published and in this study we compared these recommendations with content from the post-graduate training scheme in Geriatric Medicine in Ireland.

**Methods:**

We examined the content of didactic study-day lectures delivered during Geriatric medicine training in Ireland. We compared how both the formal Irish curriculum and the content of the study days match up with the 36 items that are identified as core knowledge content areas.

**Results:**

The Irish geriatric medicine curriculum outlined that 30 of the 36 knowledge areas from the European curriculum should be covered. Formal teaching was delivered on 33 of the 36 knowledge components that are outlined in the European curriculum. 24 of 36 topics were covered at least twice.

**Conclusion:**

There was a high concordance between the content of the Irish and European post-graduate curriculum in Geriatric medicine.

## Background

In 2019, the European postgraduate curriculum in Geriatric Medicine outlined the minimum recommended training requirements to become a geriatrician at the specialist level in the EU [1]. This framework for education and training identified four different domains that are endorsed as minimum training requirements to become a geriatrician at specialist levels in EU member states. The knowledge section of the curriculum identifies 36 sub-items that were deemed essential after a modified Delphi technique. This curriculum follows from the earlier development of European recommendations for undergraduate training in Geriatric medicine [2], with the undergraduate curriculum having been successfully adopted across different European universities [3, 4].

It is inherently more difficult to compare and contrast the content and delivery of post-graduate curricula over under-graduate curricula [5]. In this study, we systematically studied the content of didactic Irish higher specialist training (HST) Geriatric medicine study days and compared how the content of the Irish curriculum benchmarks against the knowledge content recommendations from the European post-graduate curriculum in Geriatric medicine.

## Methods

The HST in Geriatric Medicine is a 5 year, postgraduate, training scheme with a curriculum approved by the Royal College of Physicians of Ireland to be covered during this training [6]. Structured learning opportunities throughout the training scheme are delivered through a combination of required courses, masterclasses, departmental educational sessions (such as journal clubs and grand rounds), and through attendance at specific Geriatric medicine study days. Trainees are required to attend a minimum of 75% of the Geriatric medicine study days per training year. These study days rotate across different training sites, and a list of suggested talks to be covered is provided by the National Specialty Directors to each site in advance of a study day. In this study, we examined the content of didactic lectures given over a 5 year, rolling, time-period (the duration of HST in Geriatric medicine in Ireland), with content determined by thematic analysis of the content of individual lectures across the study days. We compared how the content corresponds to the knowledge content recommendations present in the European post-graduate curriculum in Geriatric medicine. If the study day contained content outside the knowledge recommendations, more relevant to the local framework, this content was labelled as “Other”.

## Results

There were 24 study days completed over the 5 year period. In the first 3 years of this 5 year period there were four geriatric medicine study days planned per year, and for the final 2 years there were seven geriatric medicine study days per year. During the third year of this time-period, two of the study days were cancelled at the beginning of the COVID-19 pandemic, and the remaining study days were all delivered online.

### Irish geriatric medicine curriculum

30 of the 36 European knowledge sub-items are specifically referred to in the curriculum of the Irish Geriatric Medicine HST. There was no specific reference in the Irish curriculum to sarcopenia, sleep disorders, tissue viability, iatrogenic care delivered disorders, sexuality in older adults or gerotechnology/e-health. However, despite not being specifically referred to in the Irish curriculum, there had been lectures delivered across the study days on sarcopenia, iatrogenic care delivered disorders, sexuality in older adults, and gerotechnology/e-health. Neither sleep disorders nor tissue viability were referred to in the Irish geriatric medicine curriculum, nor covered over the course of the rolling 5 year period. Pain assessment was referred to in both geriatric medicine curriculums, but not formally covered over the course of the 5 year period at study days. This meant that overall, 33 of the 36 European knowledge sub-items were specifically covered at Irish study days over the 5 year period.

### Content of study days

24 of the 36 topics were covered at least twice on study days (Table 1). Dementia was the most frequently covered topic on the different study days. This was followed by talks on the biology of ageing, acute and chronic diseases, and talks that covered malnutrition or fluid imbalance. Topics covered least commonly included dysphagia, sarcopenia, continence, depression, sexuality in older adults, comprehensive geriatric assessment, long-term care and palliative care.

**Table 1:** Items from the knowledge component of the European geriatric medicine curriculum and frequency of overlap with the Irish curriculum

0	1	2	3	4	5	6	7
Pain	Dysphagia	Falls	Parkinson’s disease	Gait disorders	Acute & chronic diseases	Biology of ageing	Dementia
Sleep disorders	Sarcopenia	Dizziness & vertigo	Stroke	Other movement disorders	Malnutrition & fluid balance		
Tissue viability	Continence	Syncope	Frailty	Pharmacological issues & ageing	Legal aspects		
	Depression	Osteoporosis	Ethical issues	Rehab & MDT care			
	Other psychiatric	Delirium	Social health inequalities				
	Sexuality in older adults	Health promotion & healthy ageing	Family caregivers				
	Comprehensive geriatric assessment	Iatrogenic care delivered disorders					
	Long term care	Gerotechnology					
	Palliative care						

## Discussion

There was considerable overlap between didactic study day lectures given over a rolling 5 year of the Irish HST programme in Geriatric medicine and the knowledge content recommendations from the European postgraduate curriculum in Geriatric Medicine. Thirty out of 36 recommended European curriculum knowledge areas were specifically referenced in the Irish HST curriculum. Thirty-three out of 36 knowledge areas were specifically covered in formal study days. This highlights that the Irish post-graduate training in Geriatric medicine is keeping pace with international recommendations for expanded education and training.

The levels of competence as well as the content required to become a Geriatrician varies considerably across various European jurisdictions [7]. Ireland’s curriculum has a solid foundation in the core knowledge competencies required for post-graduate training in Geriatric medicine. This strong benchmarking of the Irish post-graduate training curriculum has also built-in space for additional talks that align with local requirements specific to the national healthcare system. The Irish post-graduate training in Geriatric medicine complies strongly with European training standards and Irish-trained Geriatricians can encourage other EU member nations to promote Geriatric medicine as an area for sub-specialisation.

There is wide variation in the length and standard of post-graduate training programs in Geriatric medicine in Europe with the Irish geriatric medicine training being among the longest in Europe, and longer than the minimum suggested 5 years [8, 9]. It is likely that such an extended period of training allowed such a variety of topics to be covered and it is possible that countries that have considerably shorter training period may not manage to cover such depth and that concerted efforts would need to be made to ensure parity of education and training. Further international curriculum comparisons would be helpful in determining the minimum duration of training that is required to have covered the required knowledge content to be a specialist Geriatrician.

Tissue viability and sleep disorders were two subjects that did not feature in either the HST curriculum or in the content of any of the study days. Tissue viability was the area that has the lowest agreement that it should be a formal training requirement at 70% agreement in the European curriculum guidelines [1]. In contrast, sleep disorders had a higher level of agreement as a training requirement at 90%. It is likely that trainees come across sleep disturbance on a regular basis during the Geriatric medicine HST, as part of both chronic disease management and dementia care. Didactic teaching should be delivered going forward in relation to these complex subjects which are important when considering the holistic approach to caring for older adults.

A limitation of this study is that we sought to only examine the knowledge-based section of the curriculum as this is specifically taught in dedicated study days. Other aspects of the post-graduate training such as the uptake of formal post-graduate taught programmes in Geriatric medicine were not captured, nor courses such as the diploma in Medicine for the Older Person which is offered by the Royal College of Physicians of Ireland or the Irish National Frailty Education program have not been captured [10]. The Diploma in Syncope and Related Disorders is undertaken by many Irish trainees [11], and this course has structured teaching on sleep disorders, so our study does miss out on capturing other optional learning opportunities trainees avail of.

Going forward there is a need for further investment in education, to inspire the next generation of trainees and promote the development of services and societies that are responsive to the needs of all older adults (12). Our study highlights that Irish trainees in Geriatric medicine are being prepared to meet such demands.

## Data Availability

Data that supports the findings is available upon request from the corresponding author (RM).
